# Cell plasticity modulation by flavonoids in resistant breast carcinoma targeting the nuclear factor kappa B signaling

**DOI:** 10.1007/s10555-023-10134-x

**Published:** 2023-10-04

**Authors:** Peter Kubatka, Lenka Koklesova, Alena Mazurakova, Aranka Brockmueller, Dietrich Büsselberg, Martin Kello, Mehdi Shakibaei

**Affiliations:** 1 Department of Histology and Embryology, Jessenius Faculty of Medicine, Comenius University in Bratislava, Martin, Slovakia; 2 Clinic of Obstetrics and Gynecology, Jessenius Faculty of Medicine, Comenius University in Bratislava, Martin, Slovakia; 3 Department of Anatomy, Jessenius Faculty of Medicine, Comenius University in Bratislava, Martin, Slovakia; 4 Chair of Vegetative Anatomy, Institute of Anatomy, Faculty of Medicine, LMU Munich, Pettenkoferstr. 11, D-80336, Munich, Germany; 5 Department of Physiology and Biophysics, Weill Cornell Medicine in Qatar, Qatar Foundation, Doha, Qatar; 6 Department of Pharmacology, Faculty of Medicine, Pavol Jozef Safarik University, Kosice, Slovakia

**Keywords:** Breast carcinoma, Chemo-resistance of cancer, Cell plasticity, NF-κB signaling, Flavonoids, Combination anti-cancer therapies

## Abstract

Cancer cell plasticity plays a crucial role in tumor initiation, progression, and metastasis and is implicated in the multiple cancer defense mechanisms associated with therapy resistance and therapy evasion. Cancer resistance represents one of the significant obstacles in the clinical management of cancer. Some reversal chemosensitizing agents have been developed to resolve this serious clinical problem, but they have not yet been proven applicable in oncological practice. Activated nuclear factor kappa B (NF-κB) is a frequently observed biomarker in chemoresistant breast cancer (BC). Therefore, it denotes an attractive cellular target to mitigate cancer resistance. We summarize that flavonoids represent an essential class of phytochemicals that act as significant regulators of NF-κB signaling and negatively affect the fundamental cellular processes contributing to acquired cell plasticity and drug resistance. In this regard, flavokawain A, icariin, alpinetin, genistein, wogonin, apigenin, oroxylin A, xanthohumol, EGCG, hesperidin, naringenin, orientin, luteolin, delphinidin, fisetin, norwogonin, curcumin, cardamonin, methyl gallate and catechin-3-O-gallate, ampelopsin, puerarin, hyperoside, baicalein, paratocarpin E, and kaempferol and also synthetic flavonoids such as LFG-500 and 5,3′-dihydroxy-3,6,7,8,4′-pentamethoxyflavone have been reported to specifically interfere with the NF-κB pathway with complex signaling consequences in BC cells and could be potentially crucial in re-sensitizing unresponsive BC cases. The targeting NF-κB by above-mentioned flavonoids includes the modification of tumor microenvironment and epithelial-mesenchymal transition, growth factor receptor regulations, and modulations of specific pathways such as PI3K/AKT, MAP kinase/ERK, and Janus kinase/signal transduction in BC cells. Besides that, NF-κB signaling in BC cells modulated by flavonoids has also involved the regulation of ATP-binding cassette transporters, apoptosis, autophagy, cell cycle, and changes in the activity of cancer stem cells, oncogenes, or controlling of gene repair. The evaluation of conventional therapies in combination with plasticity-regulating/sensitizing agents offers new opportunities to make significant progress towards a complete cure for cancer.

**Graphical abstract:**

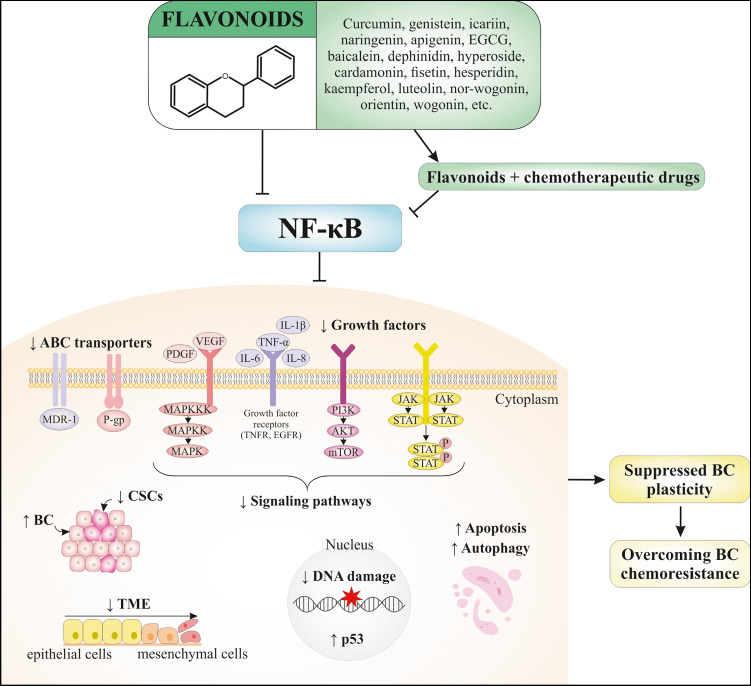

## Introduction

Impaired intracellular signaling may affect all mechanisms related to carcinogenesis, such as apoptosis resistance, excessive cell proliferation, angiogenesis, invasion, metastasis, cancer stem cell viability, plasticity, or chemotherapeutic resistance [1]. In this context, upregulated nuclear factor kappa B (NF-κB) provides transcriptional and phenotypic plasticity to a cell, thereby bringing deep local modulations in the composition and functions of a specific tissue. Based on comprehensive preclinical data, NF-κB dependent mechanisms of intra-clonal signaling inducing cancer cell plasticity lead to the acquisition of cancer-aggressive characteristics [2–4]. NF-κB signaling *via* expression regulation of multiple target genes (e.g., *BCLXL*, *BCL2*, *BCLXS*, *TNF-α*, *IL6*, *XIAP*, and *VEGF*) represents an essential cellular pathway that is extensively involved in acquired therapy resistance in cancer [5]. In many cases, cells evade anti-cancer therapies and relapse *via* phenotypic switching triggered by induced cell plasticity [4]. It includes processes such as cellular dedifferentiation, transdifferentiation, or transdetermination. Several important therapy-induced cancer cell plasticity mechanisms have been observed, including activation/suppression of key signaling pathways, modulation of transcription factors, and modulation of the tumor microenvironment (TME). Besides that, epithelial-mesenchymal transition (EMT), cancer stem cell (CSC) formation, aberrant ATP-binding cassette (ABC) transporters, and also deregulated apoptosis, autophagy, cell cycle, and mutagenesis represent crucial linkage towards therapy-induced tumor cell plasticity [4, 6, 7].

The dynamics of cellular resistance to chemotherapy in tumor cells can be either intrinsic (tumor cells are already insensitive before the respective chemotherapy) or acquired (insensitivity is acquired only during chemotherapy). Despite distinct progress in anti-cancer therapies, developing acquired drug resistance remains the crucial problem of clinical oncology and the leading cause of cancer-related mortality. Acquired drug resistance develops after the initial anti-cancer treatment and means a gradual decrease in therapy efficacy [8], resulting from several mechanisms, such as the upregulation of specific driver genes, accumulation of mutations with modified expression of molecular targets, or alterations in the TME. Data from experimental oncology found that often-used chemotherapy drugs, such as platinum-based agents, taxanes, or anthracyclines, upregulate the NF-κB signaling pathway [9, 10]. A large body of evidence has supported that systemic phosphorylation and stimulation of NF-κB prevents tumor cell death and forms drug resistance induced by various conventional chemotherapeutics [11–14] and is thus associated with an unfavorable prognosis of tumor recovery.

For the above-mentioned reason, NF-κB has emerged as a promising target for developing novel anti-cancer therapies against breast carcinoma (BC) [15]. To deceive BC resistance, several reversal chemotherapeutic agents have been introduced in clinical research, but most of them were not applicable due to severely undesirable effects in cancer patients or repeated resistance occurring in a short time [16, 17]. On the other hand, specific plant-derived molecules or plant extracts have been described to overcome multidrug resistance (MDR) in BC, including flavonoids [18–20]. The simultaneous treatment of standard anti-BC drugs and plant-derived substances represents a rational clinical approach to avoid the potential loss of effectiveness using conventional chemotherapeutic agents alone [21].

Flavonoids, of which over 8000 molecules are known, are abundant in plants, foods, and herbs. As multi-target components, flavonoids show several beneficial activities, such as anti-inflammatory and anti-cancer [18, 21–35], as well as modulate the plasticity of cancer cells with the ability to reverse tumor resistance to chemotherapeutic agents while exhibiting very low toxicity and very high efficacy [36]. They possess a broad spectrum of anticancer activities, including suppressing NF-κB triggered signaling [18]. Such modulation includes several mechanisms such as suppressing kinase phosphorylation, stabilization of IκB, an inhibitor of NF-κB, blocking interactions between DNA and NF-κB, or suppressing the nuclear translocation of NF-κB. These changes are linked with the modulation of BC cells’ plasticity mechanisms which include a decrease in inflammatory responses and cell survival pathways [37]. These and other research data provide a clear background for further exploration into flavonoids as potential molecules for the development of novel anti-cancer therapies and strategies in BC patients or effective chemopreventives [38–41].

Based on available scientific data, we are not aware of a review publication that would comprehensively summarize the role of flavonoids in the modulation of cell plasticity in resistant BC targeting the NF-κB signaling. For this reason, this review is aimed at flavonoids’ activities to modulate cancer cell plasticity towards increasing the sensitivity of BC cells to conventional treatment modalities by affecting the NF-κB-linked signaling pathways. The rational design of combination anti-cancer therapy using flavonoids is warranted to improve the clinical outcome of oncology patients. Research approaches must be addressed to the elucidation of mechanisms, which can sensitize drug-resistant cell lines to chemotherapy and hinder the initiation of drug resistance and the development of cancer progenitor cells by epigenetic mechanisms, as well as suppress the formation of cancer progenitor cells that are insensitive to conventional anti-cancer therapies [42]. Well-described oncostatic activities of flavonoids documented within preclinical research are considered of particularly great clinical meaning when administered in combination with traditional anti-cancer therapies tailored to the personalized profile of an individual [43–45].

### Source of the analyzed research data, inclusion, and exclusion criteria

Data were collected from the PubMed database sources utilizing “breast carcinoma” and “cell plasticity,” and “resistance” and “flavonoids” or “flavanones” or “flavonols” or “flavones” or “flavanols” or “isoflavonoids” or “chalcones” or “anthocyanidins” and “radiotherapy” or “chemotherapy” or “targeted therapy” or other relevant items as either keywords or medical subject heading (MeSH) terms.

The inclusion criteria used in special Chapter 3 were as follows: (a) studies showing the effect of flavonoids in the BC; (b) the experimental group was administrated with nanomaterials and any kind of flavonoids; (c) effects of flavonoids were associated with the modulation of NF-κB signaling; (d) studies *in vitro*, animal, and human using natural or synthetic flavonoids; (e) controlled experiments; (f) studies with pure flavonoids and combination therapies; (g) studies reporting the modulation of cancer cell plasticity and/or overcoming drug chemoresistance by flavonoids via NF-κB signaling.

The exclusion criteria applied in Chapter 3 were as follows: (a) non-original full research articles; (b) phytochemicals` interventions different from flavonoids or without precise dose and duration of administration; (c) interventions with plant extracts and herbals in which flavonoids were included; (d) studies reporting the use of flavonoids in the treatment of other tumors than BC or modulation of other signaling than NF-κB.

## The NF-κB signaling-induced cell plasticity and the development of BC chemotherapeutic resistance

### NF-κB and BC

NF-κB is found in most cell types and regulates genes with different functions, and is present in numerous cellular responses to various stimuli such as cytokines, free radicals, various xenobiotics, heavy metals, oxidized LDL, ultraviolet irradiation, and viral or bacterial infections/antigens [46]. In this regard, NF-κB regulates more than 500 genes, which affect processes strongly associated with all stages of carcinogenesis [47]. Except for inflammation, it includes cellular transformation, survival, proliferation, angiogenesis, invasion, and metastasis. In normal and quiescent cells, NF-κB proteins (p65/RelA, RelB, c-Rel, NF-κB1/p50, and NF-κB2/p52), as transcription factors are held back in the cytoplasm of cells by specific inhibitory IκB proteins (IκBα, IκBβ, and IκBε) [48–51]. All members of the NF-κB family (except RelB) can homodimerize and heterodimerize with each other. The dominant form of activated NF-κB is a heterodimer consisting of a p50 or p52 subunit and p65, containing transactivation domains crucial for the expression of gene information [52]. Moreover, when NF-κB is sufficiently activated/phosphorylated (e.g., by TME, pro-inflammatory cytokines, bacterial and other products, chemotherapeutic agents, and radiation), this occurs either via the canonical (classical) pathway or via a non-canonical (alternative) pathway that is induced by NF-κB proteins.

**Fig. 1:**
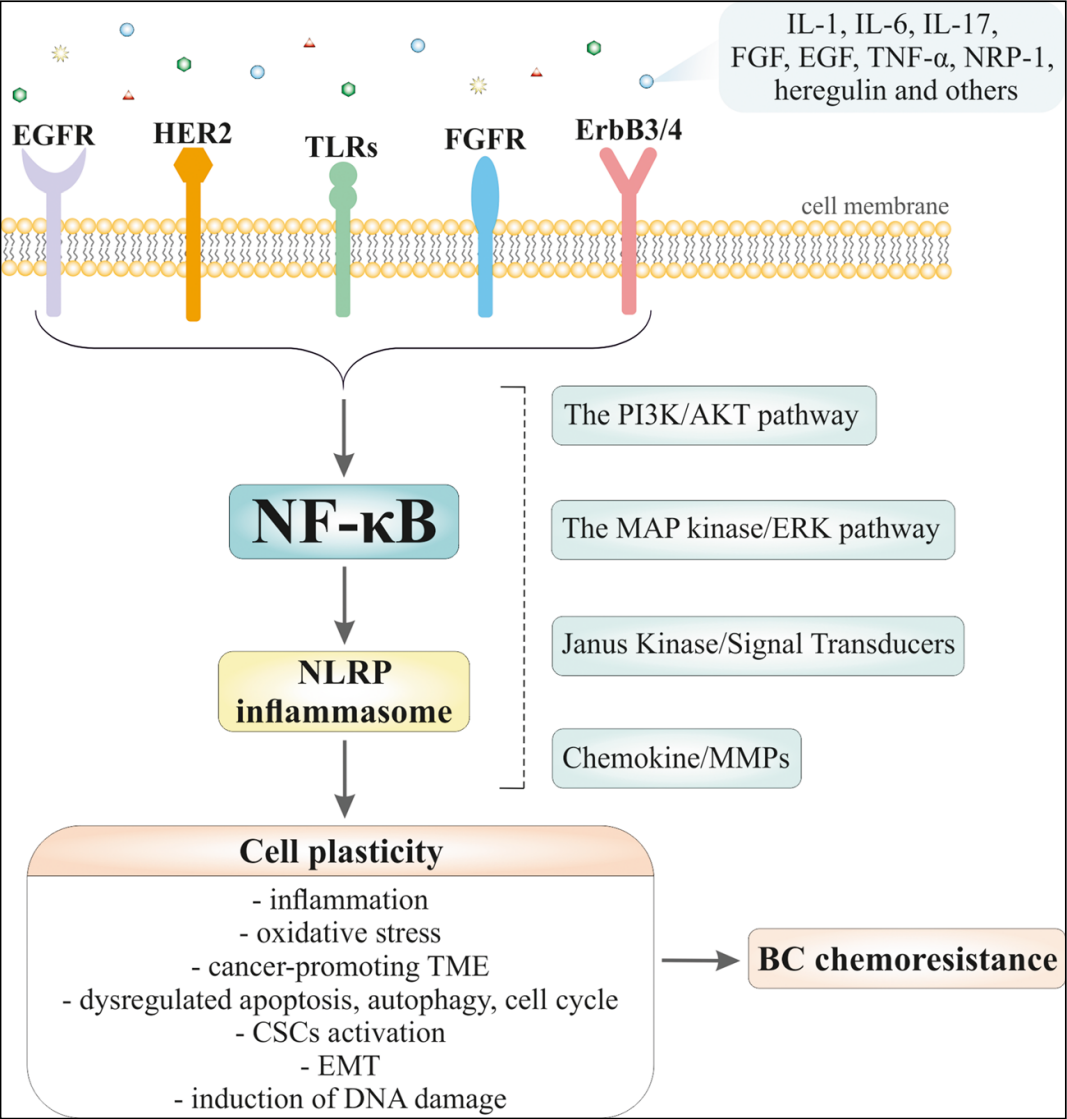
Molecular mechanisms of the cancer cell plasticity induction and BC chemoresistance development modulated via NF-κB signaling. Numerous signal molecules and membrane receptors (e.g., EGFR, HER2, TLRs, FGFR, and ErbB3/4) are included in the activation of NF-κB. Consequently, NF-κB via various signaling pathways, such as the PI3K/AKT, the MAP kinase/ERK, JAK, and chemokine/MMPs, modulates plasticity of cancer cells which includes multiple mechanisms such as inflammation, oxidative stress, TME, cell death, cell cycle, CSCs, EMT, and DNA damage. Abbreviations: CSCs, cancer stem cells; IL, interleukin; EGF, epidermal growth factor; FGF, fibroblastic growth factor; HER2 (neu/erb-b2), human epidermal receptor-2; JAK, Janus kinase; Nrp1, neuropilin-1; TME, tumor microenvironment; TNF, tumor necrosis factor

**Fig. 2:**
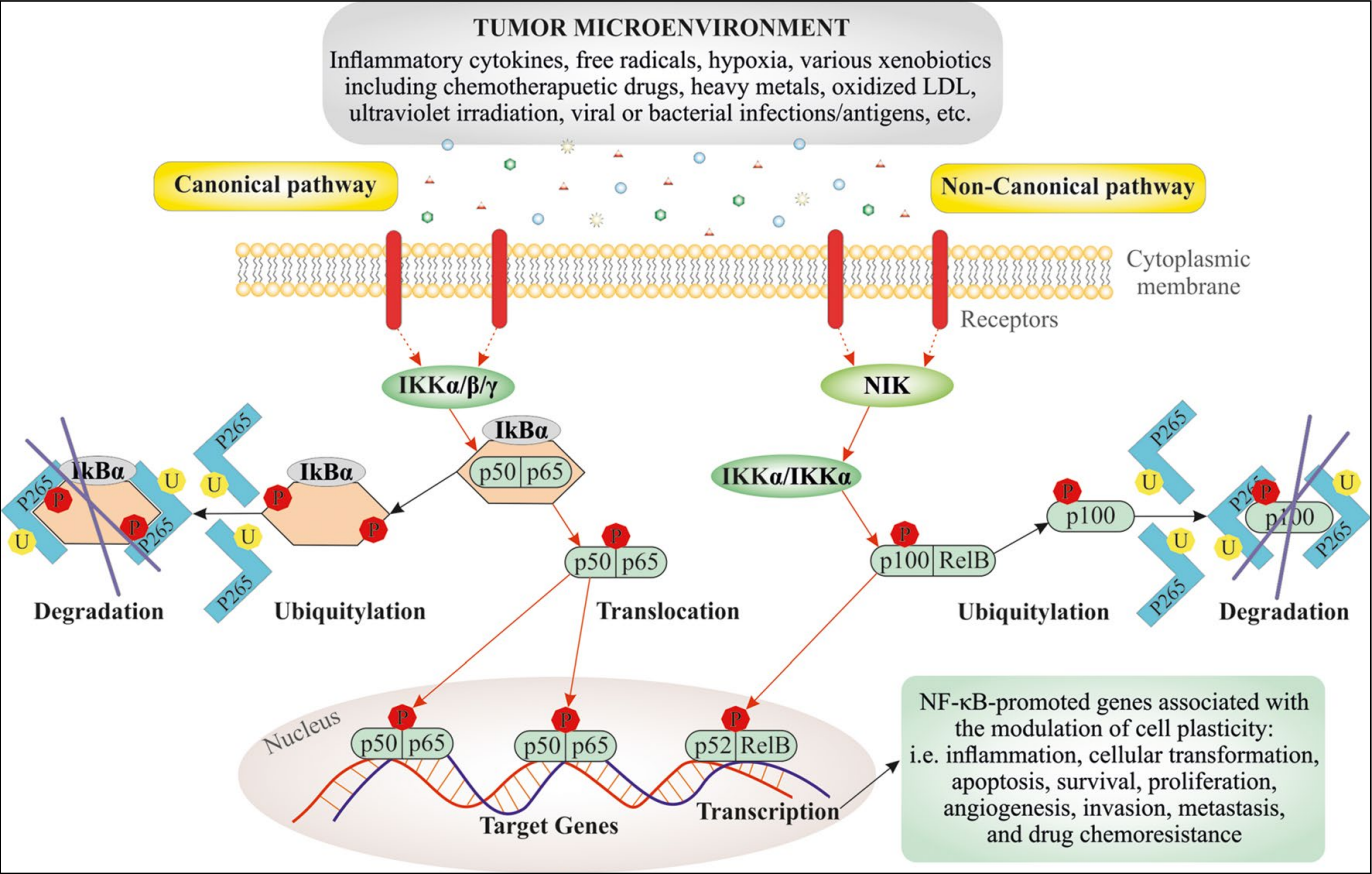
Graphical representation of major NF-κB action pathways in cancer cells arising in the TME. The canonical pathway is triggered by numerous signals, e.g., proinflammatory cytokines, xenobiotics, or viral and bacterial infections. After activation of the corresponding receptors, the IKK complexes (α and β) are activated, then IKK-mediated IκBα-phosphorylation and subsequent degradation are initiated, leading to rapid and transient nuclear translocation of the prototypical NF-κB heterodimer p56/p50. The non-canonical NF-κB pathway is activated by various ligands such as RANKL, BAFF, and CD40L. In response to NIK, IKKα is phosphorylated, which then converts p100 to mature p52. The p52 then migrates to the nucleus via its dimerization with RelB to activate non-canonical NF-κB target genes. NF-κB regulates processes associated with all stages of carcinogenesis including modulation of cell plasticity, i.e., inflammation, cellular transformation, apoptosis, survival, proliferation, angiogenesis, invasion, metastasis, and drug chemoresistance. Abbreviations: P26S, 26S proteasome; IκBα, an inhibitor of kappa B α; EMT, epithelial-mesenchymal transition; NIK, NF-κB-inducing kinase; RANKL, receptor activator of NF-κB ligand; BAFF, B-cell activating factor; CD40L, CD40 ligand

The former pathway modulates inflammatory responses, and the latter is included in immune cell differentiation/maturation and secondary lymphoid organogenesis [53]. While the canonical NF-κB signaling depends on a series of signaling cascades culminating in the activation of the IκB kinase (IKK) complex comprising IKK𝛼 (IKK1), IKK𝛽 (IKK2), and IKK𝛾 (NEMO), the non-canonical NF-κB pathway does not require mentioned IKK components instead of phosphorylated IKK𝛼. More specifically, the canonical pathway of NF-κB activation proceeds in numerous discrete steps involving phosphorylation, ubiquitination, and subsequent degradation of the specific NF-κB blocker (IκBα), which contributes to the translocation of the released subunits (p50/p65) of NF-κB to the nucleus, in turn triggering phosphorylation, acetylation, and methylation of p65 unit, followed by linkage to DNA, and then target gene transcription (Figs. 1 and 2) [48, 54]. On the other hand, non-canonical NF-κB signaling is slow and responds to a different set of ligands, such as CD40 ligand (CD40L), receptor activator of NF-κB ligand (RANKL), tumor necrosis factor (TNF)-like weak inducer of apoptosis (TWEAK), B cell-activating factor (BAFF), lymphotoxin beta (LTβ), and TNFSF14 TNF superfamily member 14 (LIGHT) leading to phosphorylation of IKKα by NF-κB-inducing kinase (NIK), which converts p100 to mature p52. The p52 then migrates to the nucleus via its dimerization with RelB to activate non-canonical NF-κB target genes [55].

BC is among the most frequent cancer diagnoses and the leading cause of cancer-related deaths in women worldwide [56–58]. In this regard, NF-κB represents a crucial transcription factor involved in the physiologic morphogenesis of the mammary gland [59]. However, dysregulated constitutive expression of NF-κB subunits (such as c-Rel, p50, and p65) has been described as a commonly observed molecular marker in BC patients [9, 60–64]. Activation of NF-κB signal machinery correlates with increased BC development and progression [65]. More specifically, a constitutive increase in NF-κB expression is significantly associated with the aggressive biological behavior of BC. NF-κB overexpression positively correlates with the larger tumor volume, negative estrogen/progesterone receptor status, and overexpressed c-erbB2 oncoprotein [62].

The deregulation of NF-κB activity causes the persistent nuclear localization of other NF-κB protein subunits such as p50, p52, cRel, and RelB. It leads to a dysbalance between apoptotic and cell cycle processes via the induced overexpression of anti-apoptotic regulators [61]. In addition, upregulated NF-κB signaling alone or in combination with other signaling pathways elevates other processes associated with BC aggressiveness and therapy resistance, such as the EMT, neovascularization, and increase in cancer cell “stemness” [7, 66]. Finally, the upregulated activity of NF-κB is strongly associated with invasive BC phenotypes that are linked with clinically relevant events such as advanced forms of the disease, early relapse, and decreased overall patient survival [3].

NF-κB signaling is of unusual importance in breast carcinogenesis [67, 68]. Constitutive activation of NF-κB signaling promotes the proliferation of high-grade, invasive, hormone-independent, and late-stage cancer phenotypes [63]. Cellular plasticity is a fundamental characteristic of cells. The mechanisms linked with cellular plasticity are reversible, and the phenotypic variations can be explicitly directed in the tissue [4]. The standardly applied cancer therapies activate the NF-κB pathway, forming invasive BCs that demonstrate resistance to various treatments, including chemotherapy, radiotherapy, and endocrine drugs. In this regard, NF-κB signaling seems to have a crucial role in BC resistance [69].

### Strategies of chemoresistance formation in BC cells

Many chemotherapeutic drugs that are usually employed for managing BC, such as mitomycin C, paclitaxel, doxorubicin, and docetaxel, are not efficacious in the clinical setting due to the development of a chemoresistance [70–73]. The main mechanisms of resistance formation (intrinsic and acquired) are explained below.

#### Cancer drug uptake, outflow, and chemoresistance

Considering that the chemotherapeutic drug must enter the intracellular space of tumor cells sufficiently to exert its actions, any irregularity in the uptake or outflow of the anti-cancer drug could be one of the most important causes of the active pathway of anti-cancer drug resistance development [74, 75]. In addition, drug outflow from tumor cells is ensured by the presence of so-called transporter proteins known as ATP-dependent multidrug carriers. The major drug transporters associated with the acquisition of drug resistance in tumors include MDR protein [74], multidrug resistance linked protein1 (MRP1), and BC resistance protein (BCRP) [76].

#### Inhibitory effect of anti-cancer drugs and chemoresistance

There is another mechanism of anti-cancer drug resistance that is favored by the glutathione S-transferase (GST) system. Namely, there is a correlation between tumor resistance and a significant increase in the GST system [77]. In addition, the enzyme GST-P1 is significantly and frequently involved in modifying anti-cancer drugs with lower efficacy and drug inactivation. It thus may be responsible for the chemoresistance against anti-cancer medications [78, 79].

### Role of NF-κB signaling in cell plasticity and chemoresistant BC development

Extensive oncology research has documented several key mechanisms and signaling pathways associated with NF-κB activation and the resulting chemoresistance of BC.

#### Tumor microenvironment

It is generally accepted that tumors can become resistant to conventional clinical chemotherapeutic agents through a number of processes. Indeed, studies have shown that the specific pro-inflammatory TME, of which BC is also a part, plays a crucial role in this process [80–82]. Chronic inflammation modulates the activity of specific enzymes, which trigger NF-κB signaling and induce robust alterations in the chromatin functions. Such modifications were induced by NF-κB support cellular plasticity via cell-specific gene expression involving the altered activity of various cytokines and adhesion factors and changes in the cell response to hormones [83].

The phenotypic plasticity of cancer cells permits deep local alterations in tissue functions. In this regard, the TME with its particular composition of cellular components, such as tumor cells, cancer-associated fibroblasts, lymphocytes, endothelial cells, mesenchymal stem cells, tumor-associated macrophages, and non-cellular components, such as pro-inflammatory cytokines, such as interleukin (IL)-1β, TNF-α, TNF-β, extracellular matrix (ECM), transforming growth factor beta (TGF-β), hypoxia-inducible factor (HIF)-1α, as well as the interaction between the cells and the surrounding matrix by specific receptors, such as integrins, are of crucial central importance for further tumor development, including epithelial to EMT, which gives the cells the capabilities to become metastatic and aggressive [84–87]. In this regard, the aggressive phenotype of BC strongly correlates with the expression of the EMT transcription factors (EMT-TFs):SNAIL, SIP1, TWIST1, and SLUG [88, 89]. NF-κB and EMT-TF overactivations in cancer cells were associated with metastatic potential and drug chemoresistance. The study of Esparza-Lopez et al. (2022) demonstrated that primary BC cells with mesenchymal phenotype show activated IKK/IκBα/NF-κB signaling and upregulated Bcl-2 and Bcl-xL activities that are linked with the resistance to paclitaxel [90]. Another preclinical study found that NF-κB upregulated the expression of EMT-TF genes via its direct binding on SLUG, TWIST1, and SIP1 promoters in MDA-MB-231 BC cells [91].

Essential requirements for tumor promotion are catabolic enzymes such as matrix metalloproteinases (MMPs) [92] that are produced in the TME by tumor and stromal cells. Dysregulated MMPs trigger the degradation of ECM components in an uncontrolled manner, allowing tumor cells to start moving, which is associated with cancer metastasis to other tissues [93]. Previous studies reported a specific functional relationship between the morphological diversity of BC cells and the influence of TME. They found that chemotherapeutic agents trigger TME-induced growth and consequent chemoresistance of cancer cells instead of destroying them [80]. In addition, promotion of the NF-κB survival factor can be favored by TME factors, such as macrophage-activating B-lymphocyte-promoting TNF family ligand, T-lymphocyte-mediated CD40L transmission, and signaling via Toll-like receptors (TLRs) [94, 95], thereby promoting tumorigenesis, cell plasticity, and chemoresistance in tumor cells, including BC [96]. The administration of a drug combination using 5-fluorouracil, doxorubicin, and cyclophosphamide triggers an IL-6-dependent NF-κB signaling cascade, which promotes stemness to non-stem cancer cells and consequently induces MDR in BC [97].

These data point to the crucial role of TME in stimulating variable responses such as reprogramming metabolic and signaling pathways (e.g., NF-κB signaling) to a therapeutic extent and the development of cell plasticity and chemoresistance in cancer cells.

#### Signaling pathways

Identifying and understanding the cellular and molecular mechanisms and associated signaling pathways related to cancer cell plasticity, cancer-promoting TME, or stem cell phenotype of CSCs may reveal new treatment strategies for resistant BCs [9, 98].

##### Growth factors and growth factor receptors

The high adaptive cellular signaling plasticity and heterogeneity that includes processes such as selection pressure and clonal progression, which are induced and controlled by growth factor receptor signaling, markedly affect the development of the resistance to targeted therapy in cancer patients [99]. The IL-1 cytokine family plays a vital role in innate and adaptive immunity and wound healing. IL-1α/IL-1β cytokines promote cancer phenotypes and contribute to resistance in cancer treatment. IL-1 signaling is significantly involved in the transactivation of NF-κB [100]. There are data that IL-1/NF-κB stimulates BC via enhanced proliferation, stem cell expansion, angiogenesis, and specific hormone receptor expression, which allows BC cells to evade therapy. Similarly, external signals transmitted via IL-17RB enabled NF-κB to increase the expression of the antiapoptotic protein Bcl-2 and induced etoposide resistance in BC cells [101].

Another inflammatory cytokine-TNF-α and microbe-sensing TLRs can stimulate NF-κB signaling and thus promote cancer progression and development of cancer resistance [100]. Sanz-Moreno et al. (2021) described activating the RANKL signaling pathway raises extracellular signal-regulated kinase (ERK), NF-κB signaling, and lapatinib resistance in several human epidermal growth factor receptor 2 (HER2)-positive BC cells. On the other hand, the receptor activator of NF-κB-B (RANK) signaling deactivation sensitizes lapatinib-resistant BC cells to the drug [102]. Another study revealed that NF-κB activation is a crucial adaptive survival mechanism of cancer cells induced by the epidermal growth factor receptor (EGFR) oncogene (it is also known as an ErbB). The inhibition of this signaling provides a clinical rationale for using anti-EGFR/NF-κB co-treatment to fight residual cancer and re-sensitize cancer patient response [103, 104]. Besides that, inhibition of fibroblast growth factor receptor (FGFR)4/FRS2α-ERK1/2 signaling, which mediated NF-κB activation, decreased MDR1 expression in the doxorubicin-resistant MCF-7 cells (MCF-7/dox) [105].

Another, a HER2/NF-κB signaling activation, induces a chemo-resistant phenotype in doxorubicin-treated MCF-7 cells [106, 107]. Neuropilin-1 (Nrp1) represents a growth factor co-receptor associated with the tumorigenicity of some breast CSCs responsible for BC-resistant tumors. Glinka et al. (2012) concluded that Nrp1-induced NF-κB signaling activation has an essential role in the mammosphere constitution [108]. In another study, heregulin as a growth factor, binds to ErbB3 and ErbB4 transmembrane receptor tyrosine kinases and upregulates the expression of anti-apoptotic, invasive, and metastatic genes in BC cells via autocrine activation of NF-κB, which causes the drug-resistant phenotype of these cells [109].

##### The PI3K/AKT pathway

Phosphatidylinositol 3-kinase (PI3K) is a lipid kinase that boots signal cascades in the cell and modulates severe cytoplasmic and nuclear processes. Protein kinase B (AKT) represents a downstream effector of PI3K and regulates numerous cellular pathways associated with carcinogenesis [110]. The serine/threonine protein kinase C (PKC) has been documented to modulate c-Rel-driven BC [111]. In physiologic conditions, Forkhead box O3a (FOXO3a) binds to the Forkhead box elements in the estrogen receptor (ER) promoter. It induces expression of p27Kip1 and ERα, which represses c-Rel activity, thus preserving a normal phenotype of epithelial cells [112].

On the other hand, in malignant BC cells characterized by high mitotic index and invasive phenotype, PKC activates AKT, which consequently triggers nuclear exportation of FOXO3a. It induces decreased ERα expression and causes the re-activation of c-Rel activity. Besides PKC/AKT/FOXO3 pathway, targeting PI3K/AKT/mammalian target of rapamycin (mTOR) signaling, seems clinically essential to overcome acquired drug resistance in BC [113]. Oncology research points to the crucial role of the PI3K/AKT/mTOR pathway in drug resistance. Therefore, developing effective PI3K/AKT/mTOR inhibitors within the novel approaches to overcome acquired resistance to conventional BC therapies is necessary.

##### The RAS/MAP kinase/ERK pathway

The cell’s RAS/mitogen-activated protein kinase (MAPK)/ERK signaling represents a vital transmission pathway of various mitogen signals, including cytokines and growth factors. This pathway plays a significant role in crucial steps of carcinogenesis [114]. NF-κB overactivation is induced by a great variety of stimuli (predominantly noxious and pathogenic), among others, via stimulation of the MAPK/ERK pathway. In this regard, IkB is phosphorylated by the mitogenic-activated protein kinase MAPK or p38 [115]. The Ras-Raf-MEK-ERK signaling pathway modulates cell proliferation, differentiation, and survival.

Overexpression of individual components within the signaling cascade has been described as a typical cancer biomarker [116]. MAPK-p38 plays a vital role in inflammatory processes. At the same time, the blocking p38 activity downregulates the expression/activity of the NF-κB and decreases the expression of an NF-κB-dependent gene, inducible nitric oxide synthase (iNOS) [117].

##### The JAK/STAT pathway

Janus kinase (JAK)/signal transducer and activator of transcription (STAT) signaling represents a cornerstone in cancer progression and the development of drug resistance via two main mechanisms: as an intrinsic cancer driver of cancer proliferation and invasiveness and as a modulation of immune surveillance [118]. This pathway can be induced by various ligand molecules such as cytokines, growth factors, or hormones [119]. STAT3 can directly couple with NF-κB, and both molecules cooperatively can modulate the expression of several genes involved in cancer invasiveness and metastasis, including resistance to apoptosis [68]. Two members of the STAT family, including STAT3 and STAT5, are associated with tumor initiation and progression.

More specifically, the gene expression initiated by STAT3 has been linked with mechanisms causing the development of drug resistance, consisting of apoptotic resistance, upregulation of cancer growth, angiogenesis, invasiveness, and suppression of immune response [120]. STAT1 overexpression is also associated with resistance to chemotherapy and radiation via immunity-related guanosine triphosphatase (IRG) activation that increases cancer cell survival and causes immune exhaustion through overactivated interferon-gamma signaling [121]. However, effective STAT inhibitors in clinical practice have not been introduced. In oncology research, it is necessary to analyze also the role of small molecule inhibitors in the modulation of the JAK/STAT signaling. The effects of persistently activated JAK/STAT signaling on carcinogenesis made this pathway an important target for new drug development and effective and personalized management of BC.

#### CSCs

In fact, the prominent role of NF-κB signaling has been described for cancer stem cell activity [7, 122]. It is widely known that cancer stem cells force tumorigenesis, promote resistance to cancer therapies, and stimulate cancer recurrence, invasiveness, and metastasis through the contribution of NF-κB signaling, thereby promoting tumor cell plasticity [7]. In particular, NF-κB modulation of critical target genes, especially inhibitors of apoptotic proteins, cytokines, and EMT transcription factors, plays a critical role in the phenotype of CSC.

Regarding NF-κB activities, it also couples with other key CSC-linked signaling pathways, such as Notch, EGFR, TGF-β, and STAT3 [7, 123]. NF-κB modulates the cell cycle and colony formation of HER2-derived BC cell lines [124]. NF-κB checks the initiation of HER2 tumors via multiple signaling pathways, including cellular proliferation, vasculogenesis, inflammation, reactive oxygen species production, or invasiveness. The resistance to HER2-targeting drugs results from multiple somatic mutations, which adamantly trigger unregulated PI3K/Akt activation [125, 126] and thus support the development of chemoresistance. Besides that, IKKα is another molecule crucial for the self-renewal of cancer-initiating cells found in the HER2 BC model [124].

Espinoza-Sánchez et al. documented the ability of triple-negative breast cancer (TNBC) lines to induce CSC-like characteristics and invasiveness via a mechanism triggered by pro-inflammatory cytokines with an activated chemokine (C-X-C motif) ligand (CXCL)12/CXCR4/CXCR7 signaling pathway. The authors analyzed a CSCs- and cancer-inflammatory-linked gene crosstalk. Regulatory network assessment found an NF-κB-dependent mechanism of cell plasticity of TNBC lines associated with an aggressive phenotype transcriptional signature [3]. Finally, the RANKL/RANK/IKKα/NF-κB signaling axis represents a pathway critical for paracrine communication to stem cells [127]. The regulation of this pathway may prevent some BC cases by directing stem cells into a dormant state.

#### ABC transporters

It was found by Velaei et al. that there is a correlation between NF-κB and ABC transporters in MCF-7/Dox. Moreover, suppression of overactivated NF-κB by translocation silencing using siRNA downregulated the expression and activity of efflux pumps MDR1 and MRP1. In addition, NFκB suppression increased apoptosis by downregulating Bcl-2 and overexpressing Bax expression in MCF-7/Dox cell lines compared with MCF-7 control lines. These authors concluded that blocking the NF-κB pathway has a dual effect of enhancing apoptosis and suppressing the expression/activity of ABC transporters, which are important features of decreased cell plasticity and anticancer drug resistance [128]. Similarly, another study analyzing the same MCF-7/dox BC cells described the inhibition of MDR1 activity via the reduction of FGFR4/FRS2α-ERK1/2-induced overactivation of NF-κB signaling [105]. In addition, ABCB1 inhibition in several doxorubicin-resistant BC cell lines was associated with the downregulation of STAT3-dependent NF-κB and heat shock protein (HSP)27/p38/AKT signaling [129].

#### Apoptosis, autophagy, and cell cycle

The NF-κB pathway confers a survival advantage to cancer cells by modulating specific genes that control the balance between cell death and proliferation, thereby modulating processes of adaptive cellular plasticity of cancer cells to targeted therapies [130]. Indeed, one of the many functions of NF-κB is to increase cell survival by altering the expression of pro/anti-apoptotic genes, thereby modulating individual components of the machinery of programmed cell death in normal or cancerous cells [131]. In addition, the autophagy signaling pathway has been reported to affect the sensitivity of cancer cells to conventional cancer therapies [132]. However, NF-κB plays a dual role in autophagy in cancers; it can either support or suppress carcinogenesis, depending on the stimulus in a given tumor microenvironment [133].

The translocation blockage of STAT3 and NF-κB and their decreased transcriptional activity in the nucleus caused G2/M cell cycle arrest and increased apoptosis in doxorubicin-resistant human MCF-7 BC cells [134]. Moreover, other findings have shown that upregulation of the NF-κB/IκBα pathway may play an essential role in modulating the susceptibility of cancer to paclitaxel-induced cell cycle arrest in G2-M phase and programmed cell death [135]. Inhibition of IKK-controlled NF-κB activation provides a rational clinical strategy to overcome acquired drug chemoresistance in cancer via modulation of balance in cell death/proliferation and thus convert inflammation-induced cancer progression/resistance to cancer regression.

#### p53 oncogene

NF-κB2 signaling pathway represents one of the options through which mutant p53 cancer cells may develop cell plasticity and loss of drug sensitivity [136–138]. The correlation between p53 deficiency and activation of NF-κB was associated with decreased disease free-survival in BC individuals [139]. Aberrant p53 cancer cells stimulate TNF-α-induced NF-κB signaling, thus hindering cells from TNF-α-induced programmed cell death. In addition, mutant p53/NF-κB cross signaling enhances the proliferation of cancer cells via the MAPK-induced signaling pathways and correlates with the extent of EMT and invasiveness/metastasis [140].

Results from gain- and loss-of-function studies summarize that the anti-apoptotic effects of NF-κB signaling are constitutively stimulated by a p53 hot-spot mutation typically found in BC cells [141]. Another study revealed the correlation between p53 deficiency and NF-κB-driven doxorubicin insensitivity in BC associated with an aggressive clinical phenotype [139]. Wang et al. (2013) described that BCRP, Bcl-2, and survivin expression levels were elevated, while p53 expression was downregulated in MCF-7/5-FU cells compared to control MCF-7 cells. The authors found that survivin significantly correlated with BCRP expression. Importantly, survivin attenuated the suppression of p53 and thus increased the expression of the BCRP gene via NF-κB signaling [142].

#### DNA damage and repair mechanisms

Cellular answer to DNA damage is vital in maintaining genomic stability and survival. Defects in DNA repair processes cause multiple detrimental consequences in the cell, including cancer cell plasticity [15]. NF-κB transcription factors (along with p53) control principal responses to intracellular stress, and the DNA damage-initiated NF-κB signaling pathway has notable responsibility in developing cancer drug resistance [143]. Nucleotide excision repair (NER) activates NF-κB, and this mechanism is dependent on the ataxia telangiectasia mutated (ATM) protein [144]. ATM and rad-3-related (ATR) kinase negatively control the ATM-NF-κB signaling. Besides that, ATR suppresses the NF-κB pathway during the replicative stress by competitively binding to NF-κB essential modulator [145]. High ATR activity correlates with downregulated NF-κB pathway and elevated apoptosis in response to cisplatin treatment [146]. In addition, the same study showed that NER suppresses cisplatin-induced apoptosis via stimulation of NF-κB, which controls overexpression of the Bcl-xL gene associated with the activation of the survival pathways in fibroblasts treated with cisplatin [146]. Figure 1 summarizes different mechanisms of the development of BC chemoresistance throughout the activation of NF-κB and increased cell plasticity.

## Flavonoids as suppressors of cancer cell plasticity and consequent BC chemoresistance via NF-κB signaling

### Tumor microenvironment and EMT

A large body of evidence suggests that dysregulation of NF-κB, which is triggered and activated by TME and different pro-inflammatory cytokines, including TNF-α, TNF-β, IL-1β, growth factors, hypoxia, and others, leads to further expression and upregulation of pro-inflammatory genes, enzymes, and pro-tumorigenic proteins that may also be involved in cancer pathogenesis and development including in BC [49, 147–150]. In addition, the activated pro-survival factor NF-κB network is known to be present in most types of cancer [60, 151] and plays a critical role in many parameters of carcinogenesis such as the development of chemotherapy resistance (Fig. 2) [48–50].

Due to their versatile range of effects, flavonoids target different imbalances caused by a TME. One of the most extensive-impact strategies is the containment of inflammatory processes by inhibiting NF-κB, thereby interrupting a central cascade of BC development [152].

In addition, *in vivo* evidence for the modulation of TME by flavonoids has also been provided. For this purpose, female BALB/c mice were BC-challenged and then treated with extracts from flavokawain A, a chalcone found in the *Piper methsyticum* [153]. Besides reducing tumor volume and weight, an inhibition of NF-κB and related nitric oxide, iNOS, intercellular adhesion molecule, and cyclooxygenase 2 was observed. This finding indicates that flavonoids strengthen anti-cancerous immunity by inhibiting NF-κB in TME [153]. Furthermore, the flavonol icariin inhibits proliferation and triggers apoptosis in BC cells by downregulation of NF-κB and EMT. Moreover, icariin has a non-cytotoxic effect in normal breast cells [154]. Therefore, BC was induced in mice with MDA-MB-231 and 4T1 cell lines, and the animals were subsequently given icariin. This reduced the growth of the tumors and prevented lung metastases. Here, too, NF-κB was a central target of flavonoids in TME [154].

Other flavonoids that have shown cancer-displacing NF-κB-suppression in mouse/*in vitro* models with triple-negative BC include, for example, citrus-derived nobiletin [155] and ginger-herb alpinetin [156]. Soy-extracted genistin suppressed the activity of NF-κB and high-mobility group box1, which is profoundly involved in the beneficial remodeling of tumor/immune microenvironment [157]. In the TNBC model, wogonin caused cellular senescence in BC cells via the downregulation of thioredoxin reductase 2 gene expression and modulated NF-κB/STAT3 signaling, which is considered a regulator of senescence-associated secretory phenotype (SASP). SASP suppressed the proliferation of TNBC cells and increased polarization of macrophage M1 *in vitro* and also promoted infiltration of immune cells in a xenografted cancer model [158]. In another study, suppressed SASP induced by apigenin correlated with decreased IL-1α signaling associated with NF-κB, p38-MAPK, interleukin-1 receptor-associated kinase (IRAK)1, and IRAK4 downregulations. In addition, apigenin reduced the expression of CXCL10, a newly discovered SASP marker. Besides that, apigenin-induced suppression of the SASP secretion decreased the aggressive phenotype of human BC cells via suppressed cell growth, EMT, and ECM invasion [159].

Overall, clinical investigations focusing on this axis would be the next reasonable step after these clear indications of tumor-inhibiting NF-κB-modulation by flavonoids in TME *in vitro* and *in vivo* (Table 1).

**Table 1:**
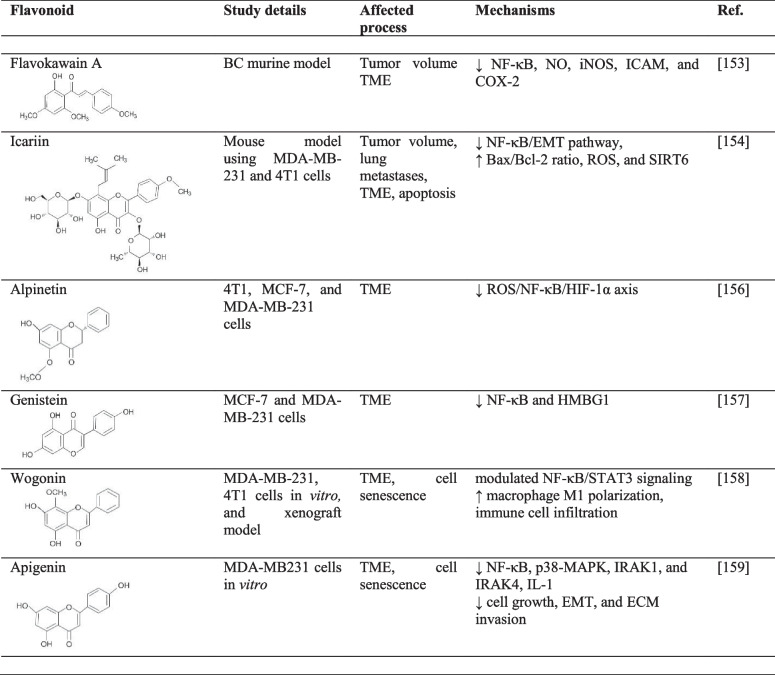
The effects of flavonoids on TME via modulation of NF-κB signaling

↓, decreased/suppressed; ↑ increased/enhanced

Abbreviations: *COX-2*, cyclooxygenase-2; *EMT*, epithelial-mesenchymal transition; *HIF-1*, hypoxia-inducible factor; *HMGB1*, high-mobility group box 1; *ICAM-1*, intercellular adhesion molecule 1; *IL*, interleukin; *IRAK*, interleukin-1 receptor-associated kinase; *MAPK*, mitogen-activated protein kinase; *NF-κB*, nuclear factor kappa-B; *NO*, nitric oxide; *NOS*, nitric oxide synthase; *ROS*, reactive oxygen species; *SIRT*, sirtuins; *STAT3*, signal transducer and activator of transcription 3; *TME*, tumor microenvironment

### Targeting NF-κB-associated signaling pathways

NF-κB takes part in several processes that cause cancer cell resistance to conventional therapies [160]. The studies below describe flavonoids’ role in chemosensitization and the suppression of chemotherapeutic drug resistance by targeting NF-κB upstream and downstream pathways (Table 2).

**Table 2:**
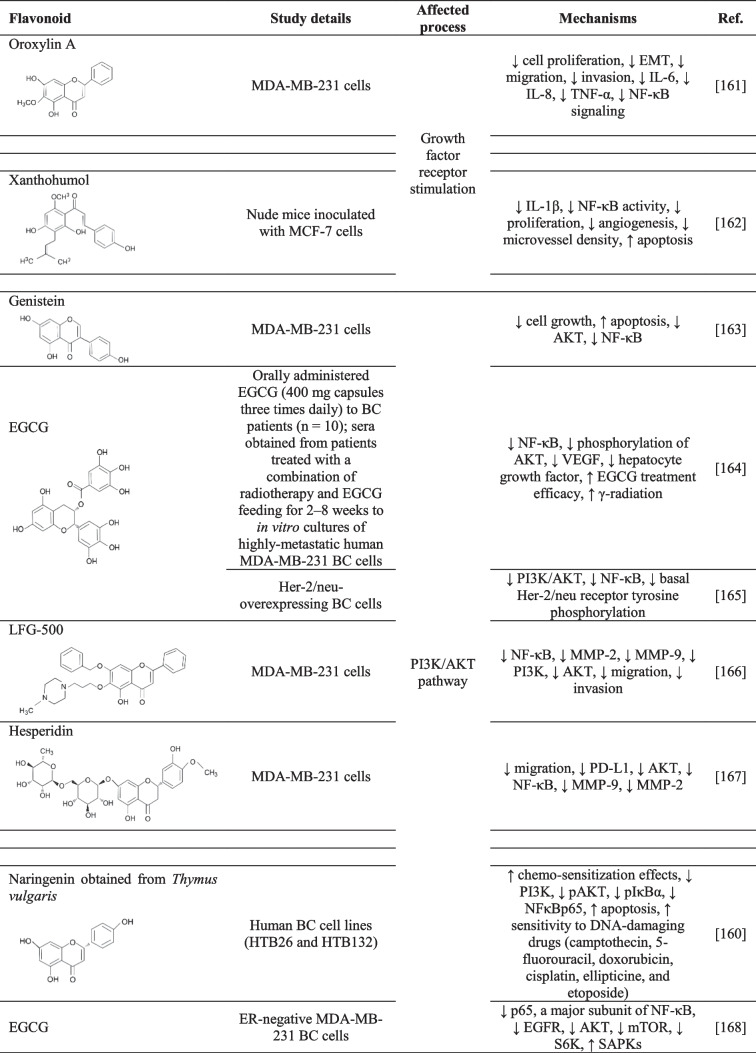

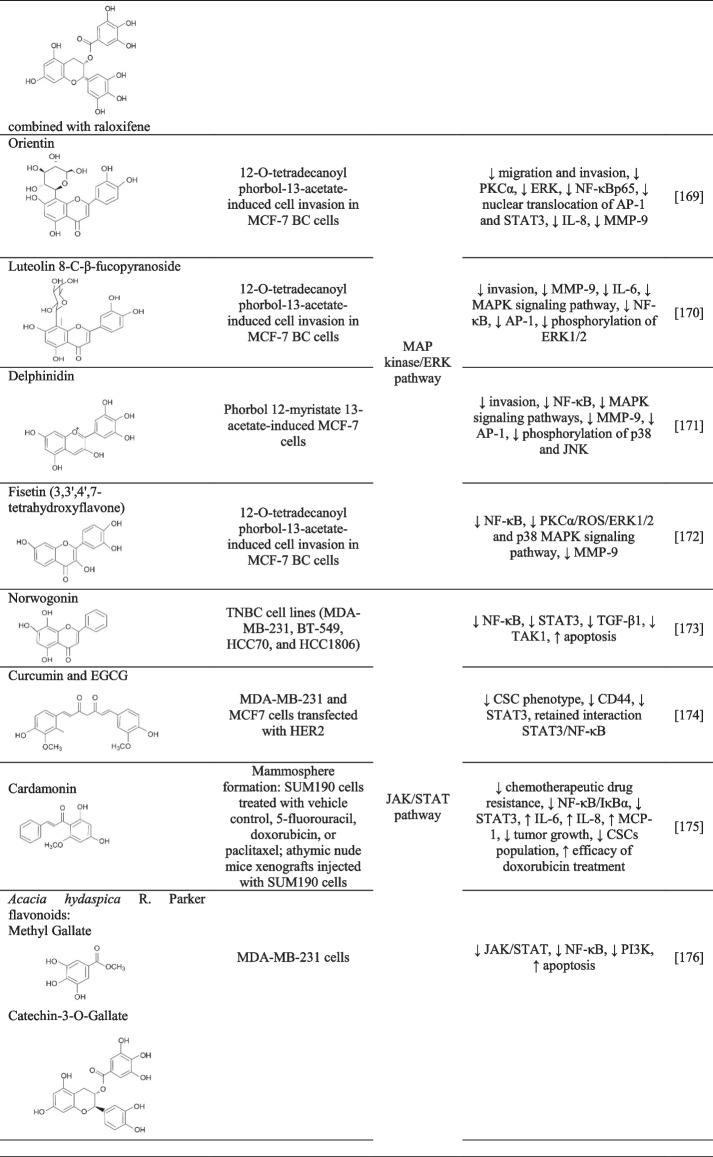
The effects of flavonoids on BC chemoresistance by targeting NF-κB upstream and downstream NF-κB signaling pathways

↓, decreased/suppressed; ↑ increased/enhanced

Abbreviations: *AKT*, protein kinase B; *AP-1*, activator protein-1; *CSC*, cancer stem cell; *DMBA*, 7, 12-dimethylbenz (a) anthracene; *EGCG*, epigallocatechin-3-gallate; *EMT*, epithelial–mesenchymal transition; *ERK*, extracellular signal-regulated kinase; *IL*, interleukin; *JNK*, c-Jun N-terminal kinase; *MCP-1*, monocyte chemoattractant protein-1; *MMP*, matrix metallopeptidase; *NF-κB*, nuclear factor kappa B; *PI3K*, phosphoinositide 3-kinase; *PKCα*, protein kinase C alpha; *p38*, mitogen-activated protein kinase; *ROS*, reactive oxygen species; *SAPKs*, stress-activated protein kinases; *STAT3*, signal transducer and activator of transcription 3; *TGF-β1*, transforming growth factor beta; *S6K*, S-6-kinase; *TAK1*, transforming growth factor-β activated kinase 1; *VEGF*, vascular endothelial growth factor

#### Growth factor receptor stimulation

Modulating the growth factor receptors that inhibit NF-κB signaling by flavonoids can lead to increased chemosensitization (Table 2). Oroxylin A, an O-methylated flavone, exerts intense anticancer activity by reversing MDR, apoptosis induction, and inhibiting angiogenesis, metastasis, and invasion [177]. In *in vitro* study, oroxylin A inhibited cell proliferation, EMT, migration, and invasion through suppressed inflammatory factors (TNF-α, IL-6, and IL-8) and suppressed the NF-κB signaling pathway in MDA-MB-231 BC cells [161]. Furthermore, *in vivo* study revealed that oral administration of xanthohumol to MCF-7 mice xenografts reduced inflammation by decreasing IL-1β, NF-κB activity, proliferation, angiogenesis, and microvessel density, and increased apoptosis [162]. The combination treatment of flavonoids along with chemotherapeutics (Table 2) can exert more efficient anti-tumor effects than the individual use of chemotherapeutics through overcoming drug resistance.

#### The PI3K/AKT pathway

Overly aggressive, invasive, and poorly differentiated TNBC (e.g., MDA-MB-231 cells) is resistant to paclitaxel and HER2- and ER-targeted treatments. The activation of NF-κB and PI3K/AKT/mTOR pathways represents the crucial mechanisms that are implicated in the survival, chemoresistance, and inhibition of chemotherapy/radiotherapy-induced apoptosis of TNBC [178–180]. This chemoresistance could be suppressed by genistein, a natural isoflavonoid from soybean products, that inhibited cell growth and triggered apoptosis via inhibited AKT and NF-κB pathways in MDA-MB-231 cells [163].

Oral administration of epigallocatechin-3-gallate (EGCG) (400 mg capsules, three times daily) to BC patients undergoing radiotherapy showed that EGCG suppressed NF-κB expression and AKT phosphorylation in the patients’ serum. In *in vitro* analysis confirmed that 5–10 μM EGCG applied to MDA-MB-231 cells enhanced the γ-radiation-induced apoptotic effects along with inhibited protein level of NF-κB and AKT phosphorylation (Table 2). In conclusion, EGCG could be an efficacy therapeutic adjuvant against human metastatic BC [164]. In another study, EGCG also reduced PI3K/AKT and NF-κB signaling pathways via inhibiting basal Her-2/neu receptor tyrosine phosphorylation in HER2/neu-overexpressing BC cells. Results suggested that EGCG could be used in adjuvant therapy, especially in tumors with HER2/neu overexpression [165].

LFG-500, a synthetically produced flavonoid, reduced MDA-MB-231 migration and invasion by decreasing NF-κB activation. Detailed analysis also revealed that LFG-500 inhibited NF-κB activation associated with PI3K/AKT but not MAPK signaling pathways [166]. In addition, programmed death ligand 1 (PD-L1) is highly overexpressed in aggressive TNBC and often causes drug resistance [181]. Hesperidin suppressed the migration of MDA-MB-231 cells through reduced PD-L1 expression that was associated with downregulated AKT and NF-κB signaling [167]. Moreover, *Thymus vulgaris* is rich in naringenin, a natural flavanone, which demonstrated chemosensitization effects on human BC (Table 2). Naringenin significantly decreased PI3K, pAKT, pIκBα, and NF-κBp65 expression levels in human BC cell lines (HTB26 and HTB132). Naringenin downregulated the PI3K/AKT pathway-increased naringenin-induced apoptosis and the sensitivity of BC cells to DNA-damaging drugs (camptothecin, 5-fluorouracil, doxorubicin, cisplatin, ellipticine, and etoposide) [160].

The combinatory treatment of EGCG and raloxifene (selective estrogen receptor modulator) reduced p65, a major subunit of NF-κB that was associated with reduced phosphorylation of the AKT, mTOR, EGFR, and S6K, and increased the phosphorylation of stress-activated protein kinases in MDA-MB-231 BC cells [168].

#### The MAP kinase/ERK pathway

MAPKs, including ERK, p38, and c-Jun N-terminal kinase (JNK), are upstream modulators of NF-κB that often cause the activation of MMP-9 expression but they are also implicated in drug resistance in carcinogenic processes [182]. Orientin, a glycosyl dietary flavonoid, inhibited the 12-O-tetradecanoyl phorbol-13-acetate (TPA)-induced cell migration and invasion in MCF-7 BC cells through reduced PKCα and ERK activation. Besides, orientin inhibited the NF-κB, the nuclear translocation of activator protein-1 (AP-1), and STAT3 and reduced expression of IL-8 and MMP-9 [169]. Similarly, luteolin 8-C-β-fucopyranoside suppressed the invasion through reduced secretion of IL-6 and MMP-9 through inhibited MAPK signaling pathway and downregulated NF-κB and AP-1 in TPA-treated MCF-7 cells. Specifically, luteolin inhibited the phosphorylation of ERK1/2, but the phosphorylation of JNK and p38 MAPK were not altered [170]. Another study revealed that delphinidin suppressed invasion via inhibited NF-κB activation and MAPK signaling pathways, especially by reduced phosphorylation of p38 and JNK, in phorbol 12-myristate 13-acetate-induced MCF-7 cell invasion. Delphinidin also inhibited AP-1 activity and MMP-9 expression [171]. Moreover, fisetin (3,3′,4′,7-tetrahydroxyflavone) reduced NF-κB activation (Table 2) and also p38 MAPK and PKCα/reactive oxygen species (ROS)/ERK1/2 signaling pathways in TPA-treated MCF-7 cells [172].

#### Janus kinase/signal transducers

The NF-κB and STAT3 pathway activation is related to TNBC cell proliferation and resistance to apoptosis. Norwogonin, a polyhydroxy flavone, inhibited the NF-κB activation and STAT3 signaling pathways linked to the suppression of transforming growth factor-β kinase 1, TGF-β1, and apoptosis induction in various TNBC cells (MDA-MB-231, HCC1806, HCC70, and BT-549 [173]. Moreover, the combinatory treatment with curcumin and EGCG suppressed the CSC phenotype, especially reduced CD44-positive cells, through inhibited STAT3 pathway in MDA-MB-231 and MCF7 cells transfected with HER2. Moreover, the interaction between STAT3 and NF-κB was retained. The results of the study can lead to the development of novel targeted therapy for treating BC against CSC-associated resistance to conventional chemo- and radiotherapy [174].

The chemotherapeutic drug resistance of breast CSCs (SUM190) could be mitigated by cardamonin that effectively reduced the activation of NF-κB/IκBα and STAT3, an essential determinant of drug resistance in BC. Moreover, *in vivo* study revealed that cardamonin in combination with chemotherapeutics (doxorubicin) prevented CSCs generation that was associated with reduced tumor growth [175]. The JAK/STAT pathway was also inhibited after the administration of polyphenolic compounds from *Acacia hydaspica* R. Parker rich in flavonoids that induced apoptosis and inhibited NF-κB and PI3K pathways in MDA-MB-231 cells [176].

### CSCs and ABC transporters

First-line chemotherapeutics are associated with the enrichment of breast CSCs. However, a flavonoid cardamonin effectively reduced breast CSCs populations enriched by first-line chemotherapeutics. At the same time, the authors highlight the potential of cardamonin to enhance the conversion of CSCs into non-CSCs. Moreover, cardamonin combined with chemotherapeutics prevented further CSCs generation, eradicated NF-κB/IκBα and STAT3 phosphorylation, and suppressed cytokines induced by therapy. Besides, NF-κB activation and STAT3 inflammatory pathways are related to the upregulation of cytokines and the associated promotion of CSCs. Also, cardamonin inhibited tumor growth and CSC pools *in vivo* [175]. Moreover, ampelopsin, also known as dihydromyricetin, inhibited stem cell properties (colony formation, mammosphere formation, CD44^+^/CD24^-/low^ populations, aldehyde dehydrogenase (ALDH) activity, and other stem cell markers such as reduced p-IκBα accompanied by suppressed NF-κB p65 among others in resistant BC cells (MDA-MB-231/IR) [183].

The *MDR1* gene, also known as *ABCB1*, encodes a multidrug transporter P-glycoprotein (P-gp), which is closely associated with cancer resistance. However, an isoflavonoid puerarin (found in *Pueraria lobata*) suppressed MDR1 at mRNA and protein levels in MDR human BC cell line (MCF-7/adr) indicating reversed MDR phenotype. The authors concluded the effects of puerarin in MDR1 suppression to be associated with the inhibition of NF-κB activation [184]. Interestingly, a nanoparticle (paclitaxel encapsulated with EGCG) sensitized paclitaxel-resistant MDA-MB-231 cells to paclitaxel (via downregulation of *ABCB1* gene and P-gp expression), induced apoptosis, and inhibited NF-κB. These results (Table 3) highlight the potential of combinatory nanomedicine in BC management [185].

**Table 3:**
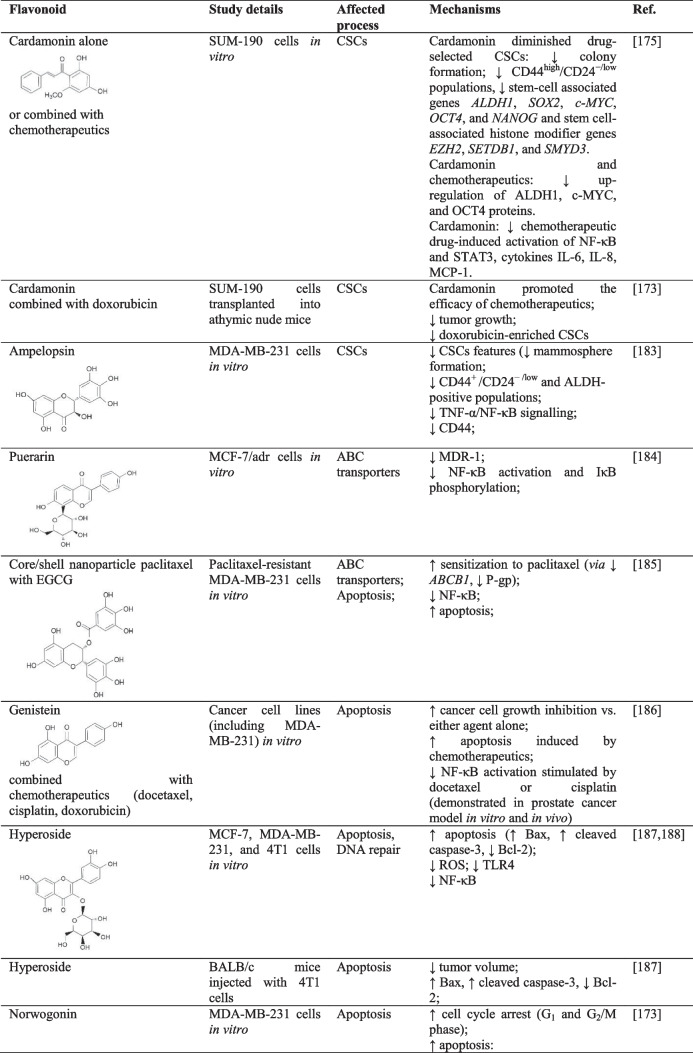

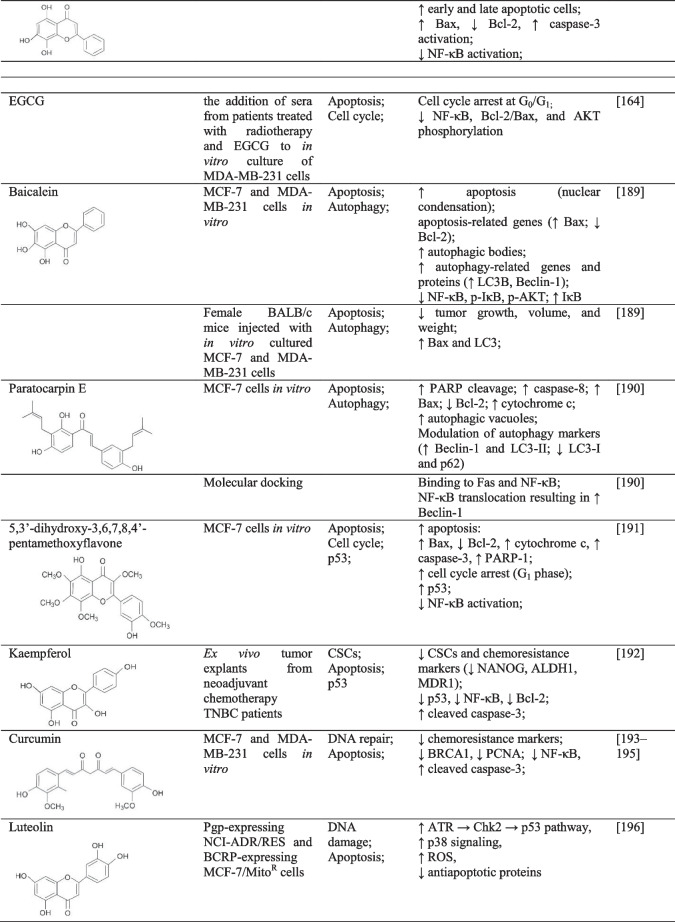
The effects of flavonoids on NF-κB-related chemoresistance of BC via different cellular mechanisms

↓, decreased/suppressed; ↑ increased/enhanced

Abbreviations: *AKT*, protein kinase B; *ALDH*, aldehyde dehydrogenase; *BRCA*, breast carcinoma gene; *CSCs*, cancer stem cells; *EGCG*, epigallocatechin-3-gallate; *IL*, interleukin; *MCP*, monocyte chemoattractant protein; *MDR*, multidrug resistance; *NANOG*, NANOG homeobox; *PARP-1*, poly [ADP-ribose] polymerase 1; *PCNA*, proliferating cell nuclear antigen; *ROS*, reactive oxygen species; *STAT*, signal transducer and activator of transcription; *TLR*, Toll-like receptor; *TNBC*, triple negative BC; *TNF*, tumor necrosis factor

### Apoptosis, cell cycle, and autophagy

The association between chemotherapy-induced NF-κB overactivation and drug resistance has been widely described [186]. As stated above, NF-κB inhibition could enhance the sensitivity of BC to chemotherapeutics and thus represents a potential strategy to overcome BC resistance [69, 197]. Anti-cancer efficacy of flavonoids, including the effects on cancer chemoresistance, is a result of the capability of natural plant substances to affect each of the multistep processes of carcinogenesis via affecting numerous signaling pathways, such as NF-κB [198, 199].

Soy isoflavone genistein has been observed to inhibit NF-κB without systemic toxicity. Specifically, in 2005, Li et al. demonstrated that pre-treatment of various cancer cells (including MDA-MB-231 cells) with genistein resulted in greater chemotherapy-induced (docetaxel, cisplatin and doxorubicin) cell growth inhibition and apoptosis when compared with genistein or a chemotherapeutic agent alone. Moreover, as further confirmed in prostate cancer cells, genistein pre-treatment effectively eliminated NF-κB-inducing activity stimulated by chemotherapeutics [186]. In addition, hyperoside, a flavonoid glycoside, promoted ROS-related apoptosis, while these effects were associated with NF-κB inhibition and Bax-caspase-3 activation *in vitro*, specifically MCF-7 and 4T1 cells. Moreover, hyperoside reduced tumor growth and modulated apoptotic parameters (Bax, Bcl-2, and caspase-3) in a subcutaneous homotransplant mouse model of BC [187]. A recent analysis (Table 3) describes the significant anti-cancer effectiveness of norwogonin, attributed explicitly to G_1_ and G_2_/M cell cycle arrest via reduced cyclin D1, cyclin B1, and cyclin-dependent kinase 1 and upregulated p21. Moreover, norwogonin induced mitochondrial apoptosis and inhibited NF-κB in TNBC cells [173].

Additionally, green tea catechins classified as flavanols are associated with potent anti-cancer activities [23, 200, 201]. Green tea catechins include flavonoids with potent biological activities, such as epicatechin, epicatechin-3-gallate, epigallocatechin, and EGCG [202]. Notably, green tea catechins have significantly inhibited carcinogenic processes in BC *in vitro* as well as *in vivo* [203]. Also, green tea polyphenols could represent potentially effective agents in enhancing the efficacy of standard therapeutic options and reducing their side effects [204]. These results, therefore, support the observations of other authors about the potent anti-cancer effectivity of whole plants or plant extracts mediated via additive or synergistic action of the mixture of phytochemicals [34, 35, 201, 205–207]. In addition, the potent anti-BC effects of green tea polyphenols have also been described in epidemiological or clinical evaluations [201]. Moreover, Zhang et al. (2012) demonstrated that flavonoids, especially green tea catechins, could potentially enhance the effectivity of radiotherapy, which is also closely associated with NF-κB signaling. Specifically, patients under radiotherapy administered also with green tea catechin EGCG showed lower expression of selected molecules associated with carcinogenesis when compared with chemotherapy alone. Moreover, adding sera from (radiotherapy and EGCG-treated) BC patients to *in vitro* culture of MDA-MB-231 cells demonstrated potent anti-cancer evidence, including G_0_/G_1_ cell cycle arrest. It reduced NF-κB, Bcl-2/Bax, and Akt phosphorylation [164].

In addition to the well-established capacity to modulate apoptosis, the broader role of NF-κB can be estimated in processes of autophagy (Table 3). NF-κB provides an advantage for cancer survival via the upregulation of anti-apoptotic genes. However, the crosstalk between NF-κB and autophagy can result either in the promotion or suppression of carcinogenesis, depending on different stimuli. Moreover, autophagy has been described as able to switch its function depending on the context. Nevertheless, as reviewed by Verzella et al., recent evidence also describes the pro-tumorigenic role of autophagy induced by NF-κB [133]. Still, current research on the effects of flavonoids on autophagy provides the following results. Baicalein increased autophagy-related genes LC3B and Beclin-1 and also induced apoptosis in BC *in vitro* and *in vivo* (specifically, MCF-7 and MDA-MB-231 cells), while the authors described also the effects on numerous signaling molecules and pathways, including downregulated NF-κB, p-IκB, or p-AKT but upregulated IκB [189]. Moreover, paratocarpin E, a chalcone widely present in *Euphorbia humifusa*, promoted autophagy (demonstrated via increased autophagic vacuoles and autophagy markers as Beclin-1) in BC MCF-7 cells. As further demonstrated in molecular docking analysis, paratocarpin E induced autophagy of BC cells through NF-κB translocation and the associated increase in Beclin-1 expression. Furthermore, paratocarpin E induced apoptosis via extrinsic as well as intrinsic pathways in the same BC cell line [190]. However, in addition to the complex association between NF-κB and autophagy, autophagy itself can also regulate NF-κB signaling [133].

### p53

p53 deficiency is associated with the inability to regulate NF-κB, promoting carcinogenesis and chemoresistance [63, 139]. Interestingly, 5,3′-dihydroxy-3,6,7,8,4′-pentamethoxyflavone is a rare flavone present in *Glycosmis ovoidea* that showed the potent capacity to inhibit NF-κB p65, increasing the activity of p53, promote the intrinsic apoptosis, and induce cell cycle arrest in MCF-7 cells [191]. Furthermore, kaempferol (Table 3) diminished the expression of stem cells and chemoresistance markers, such as NANOG homeobox protein (NANOG), ALDH1, and MDR1 accompanied by downregulated p53, NF-κB, and Bcl-2. It upregulated cleaved caspase-3 in *ex vivo* tumor explants obtained from neoadjuvant chemotherapy TNBC patients [192].

### DNA damage and repair mechanisms

As was mentioned above, the downregulation of NF-κB is beneficial in overcoming BC chemoresistance via various mechanisms. In doxorubicin-induced resistance, treatment of BC cells by curcumin reduced translocation of p65-NF-κB and thus attenuated resistance [208]. Notably, curcumin was able to downregulate DNA damage response elements (proliferating cell nuclear antigen, BRCA1) by inhibiting protein expression, transcription function, and phosphorylation of p65-NF-κB via IKK activation [193, 194]. Moreover, curcumin regulates the cellular localization of BRCA1 by triggering its cytoplasmic retention in triple-negative BC cells, preventing DNA repair, and promoting apoptosis [194, 195]. The authors concluded that curcumin’s blockage of NF-κB activity partially resensitized gemcitabine-induced chemoresistance in MDA-MB-231 cells.

Furthermore, there is evidence that another flavonoid glycoside hyperoside antagonizes BC resistance to paclitaxel. Specifically, these effects are described to be affected via the modulation of activation of pathways associated with pro-survival and inflammation mediated by TLR4. Sun et al. described that hyperoside increased the sensitivity of MDA-MB-231 cells to paclitaxel by suppressing TLR4 and p65-NF-κB expressions [188]. The apoptosis in BC induced by luteolin involves the modulation of several cellular mechanisms such as increased DNA damage, suppression of NF-κB signaling, activation of ATR → checkpoint kinase 2 → p53 pathway, activation of p38 signaling, ROS generation, and downregulation of antiapoptotic proteins [196]. The abovementioned preclinical data suggest novel clinical strategies to selectively target NF-κB and to manage BC resistance (either acquired resistance or the re-sensitization of refractory cancer cells) [143]. Based on the above-discussed data, we summarize in Table 3 evidenced effectiveness of flavonoids in regulating the processes of breast carcinogenesis and, specifically, BC chemoresistance mediated via NF-κB signaling.

## Future outlook and conclusions

Naturally occurring plant-derived molecules show significant biological activities and efficiently modulate multiple signaling pathways that are dysregulated in cancer. Preclinical research in this field points to knowledge that flavonoids represent multi-functional agents that negatively modulate most of the crucial factors contributing to MDR development and show potent capacity in increasing the chemosensitivity of cancer to conventional chemotherapeutic drugs [18]. Regarding human studies focused on reversing MDR by flavonoids, numerous of these natural agents represent active ingredients of Traditional Chinese Medicine (TCM) herb formulas [209]. TCM shows promising anti-cancer potential, including action on reversing MDR [210, 211]. For example, Yanghe Decoction is a TCM formula known for its potent efficacy in BC [212]. However, clinical trials on the analysis of pure flavonoids or flavonoid-rich formulas in BC chemo-sensitization via NF-κB signaling modulation are currently not available. Nevertheless, Zhang et al. (2022) recently introduced a randomized clinical trial to evaluate the effects of Yanghe decoction combined with neo-adjuvant chemotherapy in BC patients and the mechanism of action mediated via the PI3K/Akt/NF-κB pathway-mediated immune-inflammation microenvironment [213]. The precise clinical evaluation of anti-cancer mechanisms of pure flavonoids or flavonoid-rich herbs mediated *via* NF-κB is essential for the effective search of novel adjuvant agents with possible action on overcoming MDR within combinational strategies with conventional chemotherapeutic drugs in BC management.

**Fig. 3:**
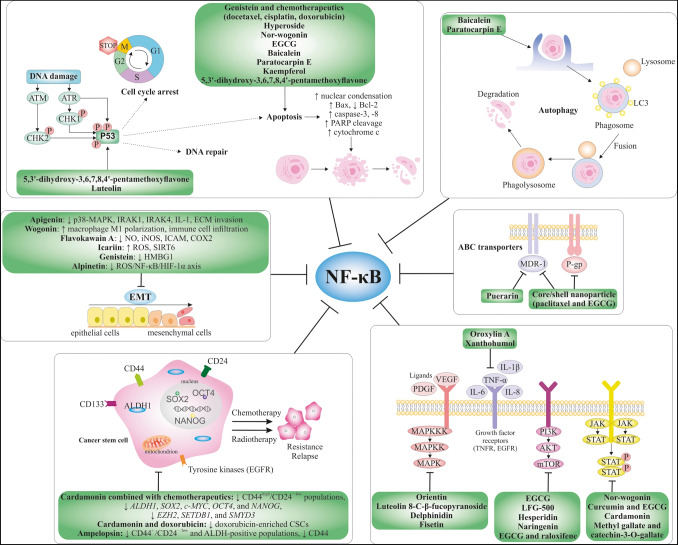
Flavonoids as potential inhibitors of the NF-κB pathway, which may act as sensitizers of the chemoresistant BC and/or chemopreventive agents. Selected flavonoids can overcome BC chemoresistance by blocking TME, ABC transporters, crucial signaling pathways, CSCs, growth factors, and DNA damage and via inducing p53 activity, apoptosis, autophagy, and cell cycle arrest. Abbreviations: BC, breast carcinoma; TME, tumor microenvironment; NF-κB, nuclear factor-kappa B; VEGF, vascular endothelial growth factor; PI3K, phosphoinositide 3-kinases; MMPs, matrix metallopeptidases; IL, interleukin; TNF-α, tumor necrosis factor-α; MDR, multi-drug resistance; MRP1, multidrug resistance-associated protein 1; AKT, protein kinase B; ERK, extracellular signal-regulated kinases; ROS, reactive oxygen species; DSB, double-strand breaks; SSB, single-strand breaks; ATR, ataxia telangiectasia and Rad3-related protein; ATM, ataxia telangiectasia mutated protein; PARP-1, poly [ADP-ribose] polymerase 1; EMT, epithelial-mesenchymal transition; ABCB1, multi-drug resistance protein 1 (MDR-1); P-gp, P-glycoprotein; CSCs, cancer stem cells; mTOR, mammalian target of rapamycin; FOXO3, forkhead box O3; JNK, c-Jun N-terminal kinase; p38, mitogen-activated protein kinase; JAK, Januse kinase; STAT, signal transducer and activator of transcription; Nrp 1, neuropilin-1; TLRs, Toll-like receptors; TGF-β, transforming growth factor beta; RANKL, receptor activator of nuclear factor-κB ligand; IKKα, IκB kinase α; OCT4, octamer-binding transcription factor 4; ALDH1, aldehyde dehydrogenase 1; SOX2, SRY-box transcription factor 2; NANOG, NANOG homeobox; CD44, cluster of differentiation 44; ABC, ATP binding cassette

In this review, we described the well-documented capability of flavonoids to modulate NF-κB signaling strongly associated with the crucial mechanisms involved in cancer cell plasticity and the consequent therapy resistance in BC (Fig. 3). Based on comprehensive data, we propose the potential implementation of flavonoids as one of the most widely occurring plant-derived substances to target NF-κB-induced cancer cell plasticity, which is related to aberrant signaling associated with crucial cellular mechanisms of BC resistance. This includes (a) modulation of growth factors and their receptors linked with the specific signaling pathways, (b) modification of transcription factors, (c) alterations in the tumor environment, (d) epithelial-mesenchymal transition, (e) cancer stem cells’ development, (f) ABC transporters, (g) modulation of apoptosis/autophagy and cancer cell proliferation, and (h) activation of gene repair processes.

Targeting the NF-κB pathway and associated upstream and downstream signaling by flavonoids has great clinical potential in the modulation of pro-inflammatory gene expressions, cancer cell plasticity, and the development of chemoresistance. Such interventions can block cancer cells from escaping immune surveillance and repress metastatic growth and cancer relapses. This knowledge shows a direction for applying innovative therapeutic strategies, including combination drug regimens focused on suppressing cancer cell plasticity.

Clinical utilization of conventional therapies combined with flavonoids as plasticity-regulating/chemosensitizing agents in the therapeutic-resistant BC represents a highly prospective approach that has to be further analyzed and optimized in preventive, predictive, and personalized oncology to achieve the goal of complete cancer cure. However, the preclinical anticancer research on the effects of flavonoids in BC cell plasticity via NF-κB signaling modulation point out to several significant limitations. The use of flavonoids as adjuvant anti-cancer drugs in human oncology is limited by their unfavorable bioavailability, complicated extraction techniques and costs, and several difficulties and obstacles found in epidemiological studies, e.g., effective use. Besides that, the targeted regulation of intestinal microflora and phase II metabolism of flavonoids can, on the other hand, modify the metabolism and toxicity of other drugs, minerals, and vitamins and thus influence the patient's health. BC research must resolve important issues such as (a) defining pharmacokinetics linked with effective and safe dosing, (b) mode of administration and new delivery systems, i.e., nano-emulsions and nanoparticles with improved target specificity and safety, (c) specification of sensitive BC types concerning individual characteristics of patients within multiomnics approach, and (d) determination of suitable combined clinical applications with conventional drugs to re-sensitize cancer cells. Future preclinical and clinical well-defined and controlled studies including the above-mentioned strategies are necessary to define whether flavonoids are suitable anti-BC drugs regarding their effectiveness, safety, and economic profitability.

## Data Availability

All data are available in the manuscript.

## Abbreviations

ABCB1: ATP-binding cassette subfamily B member 1 (also known as *MDR1*)
AKT: protein kinase B
ALDH: aldehyde dehydrogenase
AP-1: activator protein-1
ATM: ataxia telangiectasia mutated protein
ATR: Rad3-related kinase
BC: breast carcinoma
BRCA: breast carcinoma gene
BCRP: breast cancer resistance protein
CD: cluster of differentiation
CD40L: CD40 ligand
CSCs: cancer stem cells
DMBA: 7, 12-dimethylbenz (a) anthracene
MCF-7/dox: doxorubicin-resistant MCF-7 cells
EGCG: epigallocatechin-3-gallate
ECM: extracellular matrix
EGF/EGFR: epidermal growth factor receptor (receptor)
EMT: epithelial-mesenchymal transition
ER: estrogen receptor
ERK: extracellular signal-regulated kinase
FGFR: fibroblast growth factor receptor
FOXO3a: forkhead box O3a
GST: glutathione S-transferase
HER2: human epidermal growth factor receptor 2
HIF: hypoxia-inducible factor
NANOG: NANOG homeobox
HSP: heat shock protein
CXCL: chemokine (C-X-C motif) ligand
IKK: IκB-kinase
IL: interleukin
iNOS: inducible nitric oxide synthase
IRAK: interleukin-1 receptor-associated kinase
IRGs: immunity-related guanosine triphosphatases
JAK: Janus kinase
JNK: c-Jun N-terminal kinase
MAPK: mitogen-activated protein kinase
MCP-1: monocyte chemoattractant protein-1
MDR: multidrug resistance
MMP: matrix metalloproteinase
MRP1: multidrug resistance linked protein1
mTOR: mammalian target of rapamycin
NER: nucleotide excision repair
NF-κB: nuclear factor kappa B
NOS: nitric oxide synthase
Nrp1: neuropilin-1
PARP-1: poly [ADP-ribose] polymerase 1
PCNA: proliferating cell nuclear antigen
PD-L1: programmed death ligand 1
P-gp: P-glycoprotein (encoded by *MDR1*)
PI3K: phosphoinositide 3-kinase
PKC: protein kinase C
RANKL: receptor activator of nuclear factor-κB ligand
RANK: receptor activator of nuclear factor-κB
ROS: reactive oxygen species
SASP: senescence-associated secretory phenotype
SIRT: sirtuins
STAT: signal transducer and activator of transcription
TGF-β: transforming growth factor beta
TLR: Toll-like receptor
TNBC: triple-negative breast cancer
TME: tumor microenvironment
TNF: tumor necrosis factor
TPA: 12-O-tetradecanoyl phorbol-13-acetate
VEGFR2: vascular endothelial growth factor receptor 2
